# Editorial: Neuroendocrine Disorders After Traumatic Brain Injury: Past, Present and Future

**DOI:** 10.3389/fendo.2019.00386

**Published:** 2019-06-26

**Authors:** Jose M. Garcia, Ana Belen Lopez-Rodriguez

**Affiliations:** ^1^VA Puget Sound Health Care System and Division of Gerontology & Geriatric Medicine, Geriatric Research, Education and Clinical Center, University of Washington School of Medicine, Seattle, WA, United States; ^2^School of Biochemistry and Immunology, Trinity Biomedical Sciences Institute and Trinity College Institute of Neuroscience, Trinity College, Dublin, Ireland

**Keywords:** TBI (Traumatic Brain Injury), veterans, GH, pituitary hormone deficiency, IGF-I

Traumatic brain injury (TBI) has reached epidemic proportions worldwide with an increasing number of individuals being exposed as a sequela of contact sports, motor vehicle accidents or warfare. Long recognized as a potential cause for pituitary hormone deficiency (PHD), TBI is also increasingly emerging as a research priority given that, in this setting, the prevalence of PHD, the pathophysiologic mechanisms and the response to hormone replacement have not been well-established.

Several important papers published in this issue of Frontiers in Endocrinology bring new light to this area. Zhou et al. provide intriguing gene expression data for brain glucose metabolism alterations in a mouse model post-TBI that correspond with previous reports of changes in brain glucose utilization in patients post-TBI. The fact that these alterations were responsive to Telmisartan (a well-known neuroprotective agent and FDA-approved drug that was originally used to treat hypertension) opens potential therapeutic avenues that could be tested further in other animal models and, if confirmed, subsequently in human studies.

Leonhardt et al. report in a single center study that the prevalence of adult growth hormone deficiency (AGHD) using the GHRH + arginine provocative test was 12% in brain injury patients which was lower when compared with most of the previously reported cohorts. The heterogeneity (post-stroke, TBI, and subarachnoid hemorrhage patients were included in this report) and relatively small sample size were limitations of the study. However, this study also raises the importance of the provocative test used to ascertain this diagnosis as the sensitivity and specificity of different tests could vary significantly. More studies will be needed to determine the prevalence of AGHD in this setting more definitively.

Dubiel et al. performed a 6-month, randomized, placebo-controlled clinical trial of recombinant human growth hormone (rhGH) during rehabilitation from TBI in 60 individuals with mostly severe TBI. A diagnosis of AGHD was not part of the inclusion criteria for the study. The authors report that the attrition rate was low and rhGH therapy was well-tolerated. Although the rate of disability and neuropsychological function was not affected, some measures of functional independence showed a favorable trend. Whether longer treatment duration, or only limiting treatment to individuals with GH deficiency, or in the chronic phase would have resulted in a greater response remains to be determined.

Also, in this issue of Frontiers in Endocrinology, Quinn and Agha provide an excellent review of the evidence supporting the need for screening for PHD after TBI and share their clinical approach to the endocrine assessment and management of these patients. Hohl et al. review the data on acute changes in the gonadal axis, the potential mechanisms leading to these changes and their potential prognostic values of these alterations. Molaie and Maguire review the mechanisms underlying neuroendocrine abnormalities after TBI, and the potential contribution of PHD to neurobehavioral symptoms after TBI.

These reports significantly expand our knowledge about this potentially treatable complication of TBI. However, several questions remain unanswered. The relationship between severity of the injury and PHD remains to be determined. Besides, the threshold currently used for establishing the diagnosis of TBI leaves out a large number of individuals who are exposed to subconcussive injuries. Whether these patients are at risk for PHD is unknown. Autopsy data, although representing only a subset of individuals exposed, suggest that individuals exposed to subconcussive events may still experience brain damage (1). The association between the number and severity of TBIs or etiology (i.e., blunt trauma vs. blast-related) and the likelihood of PHD is also unknown. More importantly, the response to hormone replacement in this setting has been incompletely characterized and this is critical given that this patient population is very different from other patients suffering from PHD and so data from clinical trials performed in other populations cannot be necessarily extrapolated. Also, it is clear from these studies that, in order to have sufficient power to test the hypotheses posed by investigators, multicenter studies will be needed. Given that there are no effective treatments for TBI to this date, it is critical that more research is done in this area, including the development of animal models that mimic the vast spectrum of pathophysiological symptoms and psychological and behavioral sequelae after TBI as well as observational and interventional clinical trials to provide definitive answers to these questions.

## Author Contributions

JG and AL-R wrote this editorial.

### Conflict of Interest Statement

Authors received no funding from any company to write the article. JG is a consultant with Novo and has research support from Aeterna Zentaris and Pfizer. AL-R is postdoctoral in Trinity College Dublin currently funded by NIDUS. They played no role in the decision to publish, or preparation of the manuscript.
